# Effectiveness of Ecological Momentary Interventions on Pain, Mental Health, and Quality of Life in Individuals With Rheumatic Diseases: A Systematic Review and Meta-Analysis of Randomized Controlled Trials

**DOI:** 10.1155/jonm/9923240

**Published:** 2025-07-29

**Authors:** Xiaoxiao Mei, Yan Li, Wing Fai Yeung, Yule Hu, Jiaying Li, Mengqi Li, Janelle Yorke

**Affiliations:** School of Nursing, The Hong Kong Polytechnic University, Kowloon, Hong Kong, China

**Keywords:** ecological momentary intervention, mental health, meta-analysis, pain, quality of life, rheumatic diseases, systematic review

## Abstract

**Background:** Rheumatic diseases are a group of inflammatory conditions that significantly impact physical and mental health. Ecological momentary interventions have shown promising effects among individuals with rheumatic diseases as they can deliver the most appropriate type and intensity of intervention tailored to their needs in real time, but their effectiveness has not been systematically reviewed and examined.

**Objective:** To examine the effectiveness of ecological momentary interventions in reducing pain, improving mental health, and enhancing quality of life in individuals with rheumatic diseases.

**Design:** Systematic review and meta-analysis.

**Methods:** A literature search was conducted in nine electronic databases from inception to April 2024. Randomized controlled trials (RCTs) examining the impact of ecological momentary interventions on pain, mental health, and/or quality of life in rheumatic disease populations were included. Two authors independently screened studies, extracted data, and assessed the risk of bias using Cochrane's bias risk tool for randomized trials (ROB 2). The intervention effect was estimated by calculating the standardized mean difference (SMD) and 95% confidence interval (CI) with R 4.3.3 software. The certainty of the evidence was assessed using the Grading of Recommendations, Assessment, Development, and Evaluation (GRADE) approach.

**Results:** Sixteen RCTs with 1869 participants were included. Ecological momentary interventions significantly reduced pain (SMD = 0.18, 95% CI: 0.04–0.33) and marginally improved quality of life (SMD = 0.29, 95% CI: −0.01–0.60) versus controls. Subgroup analyses revealed greater effects for ecological momentary interventions with daily reminders on pain reduction (SMD = 0.21, 95% CI: 0.06–0.36) and quality-of-life improvement (SMD = 0.45, 95% CI: 0.02–0.88), as well as for ecological momentary interventions involving human contact on pain (SMD = 0.31, 95% CI: 0.10–0.52) and quality of life (SMD = 0.60, 95% CI: 0.25–0.95). However, both the primary analysis and subgroup analyses showed that ecological momentary interventions did not significantly improve anxiety and depression.

**Conclusion:** Ecological momentary interventions could alleviate pain and enhance quality of life in rheumatic disease populations, but had no significant effect on improving mental health. Future research should explore optimizing ecological momentary intervention design and implementation and further examine its effects on mental health outcomes in this population.

## 1. Introduction

Rheumatic diseases are a diverse group of inflammatory, frequently autoimmune conditions that affect the joints, muscles, bones, and organs and are associated with substantial morbidity and mortality [1, 2]. The most prevalent rheumatic diseases encompass rheumatoid arthritis, osteoarthritis, systemic lupus erythematosus, juvenile idiopathic arthritis, and gout [3, 4]. These conditions are often characterized by systemic inflammation and share common clinical features, including chronic pain, joint swelling, fatigue, and stiffness [5, 6]. These debilitating symptoms can lead to an increased risk of depression and anxiety [7–9] and significant reductions in quality of life [10, 11].

The treatment of rheumatic diseases has been revolutionized by a deeper understanding of the physiopathological connections over the last decades; however, many individuals with these conditions continue to face suboptimal management of their symptoms and ongoing challenges in their daily lives [12, 13]. The traditional models of care, which often rely on infrequent clinic visits and retrospective self-reporting of symptoms, may fail to capture the dynamic and context-dependent nature of rheumatic disease experiences [14, 15]. Moreover, these traditional models may not fully engage patients in their care or empower them to actively manage their condition between visits [16]. Consequently, there is a growing recognition of the need for more personalized, real-time approaches to symptom monitoring and intervention delivery in the management of rheumatic diseases [17, 18].

Ecological momentary interventions have emerged as a promising strategy for addressing the unique and fluctuating needs of individuals with rheumatic diseases. Ecological momentary interventions are defined as real-time interventions delivered within individuals' daily lives and natural environments, utilizing mobile technologies such as smartphones or wearable devices [19]. A key feature of ecological momentary interventions is their capacity for real-time delivery, which provides support during critical moments, such as during emotional distress or unhealthy behaviors, thus overcoming the limitations of scheduled therapy sessions [20]. Additionally, this approach utilizes data from ecological momentary assessments to tailor interventions to the user's specific needs, rather than applying a one-size-fits-all program [21]. By seamlessly integrating into the user's daily routine, ecological momentary interventions account for the individual's current environment and emotional state, thereby enhancing the relevance and effectiveness of the interventions [20, 21]. Contemporary research has begun to explore the application of ecological momentary interventions across a range of rheumatic disease populations. Several studies have investigated the use of ecological momentary interventions for pain management, with some evidence suggesting that these interventions can lead to reductions in pain intensity and improvements in pain-coping strategies among patients with systemic lupus erythematosus and osteoarthritis [22–24]. Additionally, there is an emerging literature on the use of ecological momentary interventions to support mental health [25, 26] and quality of life [27–30] in individuals with rheumatic diseases.

However, the overall effectiveness of ecological momentary interventions in this population has not been systematically reviewed and examined. Additionally, there is no summarized information about the ecological momentary intervention protocols for rheumatic diseases. As these may differ concerning their reminder strategy (e.g., daily or weekly) and supporting mechanisms (e.g., human contact or fully automated), there is a need to better describe the current ecological momentary interventions available to patients with rheumatic diseases and investigate their feasibility. Thus, the purpose of this study was to perform a systematic review and meta-analysis on the effectiveness of ecological momentary interventions in improving pain, mental health, and quality of life among individuals with rheumatic diseases.

## 2. Methods

The Preferred Reporting Items for Systematic Reviews and Meta-Analyses (PRISMA) guidelines were used when conducting and reporting this systematic review [31]. The protocol was registered in PROSPERO (International Prospective Register of Systematic Reviews; CRD42024554432). Ethical approval was not required because there was no clinical intervention performed on patients, and all data were retrieved from published articles.

### 2.1. Search Strategy for Identification of Studies

A comprehensive literature search was conducted from inception to April 2024 using the Cochrane Central Register of Controlled Trials (CENTRAL), PubMed, Embase, PsycINFO, Web of Science, Scopus, ProQuest, Wanfang Database, and China National Knowledge Infrastructure. The search strategy sample—Embase is provided in Supporting Table 1.

### 2.2. Study Selection Criteria

The articles were included based on the following PICOS criteria: (1) Population: Individuals diagnosed with rheumatic diseases; (2) Intervention: Ecological momentary intervention delivered in real-time or near real-time, utilizing data collected from specific moments in daily life, including ecological momentary assessments or a fixed timepoint obtained from individual experience [32]; (3) Comparison: No restrictions on the type of control groups, which may include attention control, standard care, routine care, or waitlist control; (4) Outcomes: Studies must quantitatively report at least one of the following outcomes: pain, quality of life, or mental health indicators (e.g., anxiety and depression); (5) Study design: RCTs or pilot RCTs.

Peer-reviewed studies published in English or Chinese were eligible, with no limitations on publication dates. Protocols, reviews, conference abstracts, and design/development papers without efficacy outcomes were excluded. Studies that used ecological momentary assessments solely for data collection or outcome measurement were also excluded.

### 2.3. Study Selection and Data Extraction

All records were imported into EndNote X9 [33]. After duplicates were removed, two reviewers (Xiaoxiao Mei and Jiaying Li) independently screened the titles and abstracts according to the established inclusion and exclusion criteria. Subsequently, a full-text assessment of potentially relevant studies was conducted by the same reviewers to identify eligible studies for inclusion in this review.

Data from the included studies were extracted independently by the same two reviewers using predesigned data collection forms guided by the Cochrane Handbook [34]. The following information was collected: (1) general information (first author, publication year, country, study design, and data analysis methods); (2) participants' characteristics (diagnosis, sample size, age, and gender distribution); (3) details of the interventions (frequency, duration, content of the ecological momentary intervention, response generation, human contact, feasibility and acceptability); (4) outcomes and measurement tools; and (5) main findings. Discrepancies were resolved through discussion and consensus, involving a third review author (L.Y.). The authors were contacted if clarification was needed.

### 2.4. Quality Assessment and Certainty of Evidence

The same two independent reviewers critically appraised the included studies using the Cochrane tool to evaluate the risk of bias (RoB 2.0) [35]. The RoB was assessed according to the following domains: (1) randomization process, (2) deviations from the intended interventions, (3) missing outcome data, (4) outcome measurement, and (5) selection of the reported result. Each domain was classified as “high RoB,” “low RoB,” or “some concerns.” The overall RoB for each outcome was judged as “low RoB” (all domains low), “some concerns” (at least one domain raised some concerns but no high risk), and “high RoB” (at least one domain high or multiple with some concerns) [35].

The overall quality of evidence was assessed using the Grading of Recommendations Assessment, Development, and Evaluation (GRADE) framework [36] by the same two reviewers. By default, the evidence of RCTs received a “high” initial grade, which was then downgraded based on prespecified criteria: RoB (assessed using RoB 2.0), inconsistency (substantial unexplained heterogeneity), indirectness (factors limiting generalizability), imprecision (considering total events/participants and 95% CI), and publication bias (evidence of small-study effects) [36].

### 2.5. Statistical Analysis

The outcomes in this study were measured using different scales; hence, standardized mean difference (SMD) and 95% confidence interval (CI) were applied. The SMD represents the difference in mean outcomes between baseline and study completion, standardized by the pooled standard deviation [37]. Given the expected variability between studies and outcome measurements, we employed a random-effects model to pool the SMDs across studies [38]. To assess the robustness of our findings, we conducted sensitivity analyses using a “leave-one-out” approach and re-estimated the overall effect [39]. This allowed us to evaluate the impact of any single study on the overall result. Subgroup analyses were performed based on reminder frequency (daily vs. weekly) and human contact involvement (yes vs. no). These subgroup analyses aimed to explore potential sources of heterogeneity and identify any differential effects across study design features. The convention proposed by Cohen [40] was used for the interpretation of the effect magnitude: trivial < 0.2, small ≥ 0.20, medium ≥ 0.50, and large ≥ 0.80. All statistical analyses were performed using the R 4.3.3 statistical software program (metaphor and meta package) [41].

## 3. Results

### 3.1. Literature Search

**Figure 1:**
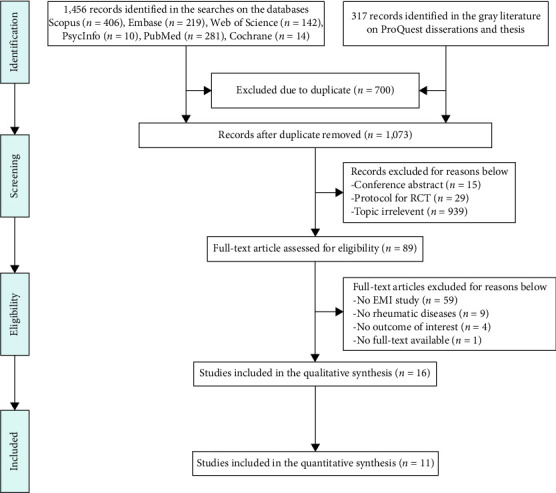
PRISMA flow diagram of the article selection process.

The database search identified 1773 studies. Following the removal of duplicates (*n* = 700), 1073 publications were screened for inclusion. Of these, 984 were excluded after reviewing the title and abstract. The remaining 89 papers were selected for full reading, and 74 were excluded because they did not include ecological momentary intervention (*n* = 59), no rheumatic diseases (*n* = 9), no outcome of interest (*n* = 4), or no full-text available (*n* = 1). Therefore, 16 studies were included in the review. However, we were unable to obtain relevant data from 5 studies [23, 27, 42–44] (i.e., data were presented graphically only or without mean difference and standard deviation, and authors did not respond to the emails soliciting the required data after multiple attempts). Therefore, 11 studies were included in the meta-analysis (Figure 1).

### 3.2. Study Characteristics

The characteristics of the included studies are shown in Table 1. Among the 16 included studies, 14 were RCTs, one was a pilot RCT, and one was reported as a pilot and feasibility RCT. In total, these studies enrolled 1869 participants, comprising 466 men and 1403 women. The study population was predominantly composed of adult participants (age > 18 years), with only one study including adolescent participants (mean age 15 years). Six studies focused on participants with knee or hip osteoarthritis, four studies included participants with rheumatoid arthritis, and three studies enrolled participants with systemic lupus erythematosus. Additionally, there was one study each that investigated participants with juvenile idiopathic arthritis and gout, and one study that included participants with either systemic lupus erythematosus or rheumatoid arthritis.

### 3.3. Intervention Characteristics

Table 2 summarizes the characteristics of the ecological momentary interventions. Overall, nine studies [22, 23, 25, 26, 29, 30, 42, 43, 45] addressed ecological momentary interventions based on self-management techniques, and four studies [44, 46–48] investigated ecological momentary interventions utilizing behavior change techniques. Two studies [27, 28] examined ecological momentary interventions grounded in an intensive treat-to-target strategy, and one study [24] explored ecological momentary interventions founded on pain-coping skills training.

The majority of studies utilized apps for participant data input [22, 24, 25, 27–30, 44, 45, 47, 48], whereas two studies employed short message service [26, 46]. Most studies adopted a daily ecological momentary assessment prompt schedule [22, 23, 25, 26, 29, 30, 42, 43, 45, 47, 48], while four studies used a weekly ecological momentary assessment prompt schedule [24, 28, 44, 46]. Only one study administered 13 text message prompts at 1- to 2-week intervals over 24 weeks [27].

Six studies utilized an artificial intelligence-based system to generate intervention responses, while 10 studies relied on pre-established rules. Besides, the ecological momentary intervention system automatically provided participants with tailored recommendations and motivational feedback without human involvement in six studies, whereas in 10 studies, researchers delivered personalized feedback and recommendations to participants using ecological momentary assessment results.

### 3.4. RoB and Certainty of Evidence

The RoB is shown in Supporting Figure 1. Overall, 13 studies were rated as having a high RoB [22–29, 43–45, 47, 48]. Ten studies presented some concerns in the randomization process [27, 44, 45] or the selection of the reported results [24, 26–30, 42, 43]. Additionally, one study presented the RoB due to missing outcome data [45]. Moreover, 13 studies were found to have a RoB in the measurement of the outcome due to a lack of blinding [22–29, 43–45, 47, 48].

From the GRADE approach, the evidence of the certainty of pain, depression, anxiety, and quality of life was graded as moderate, low, very low, and very low, respectively. The degrading factors mainly originated from the RoB of included studies, imprecision of results, and the heterogeneity across studies. The details of the GRADE evaluation are shown in Supporting Table 2.

### 3.5. Effects of Interventions

#### 3.5.1. Pain

**Figure 2:**
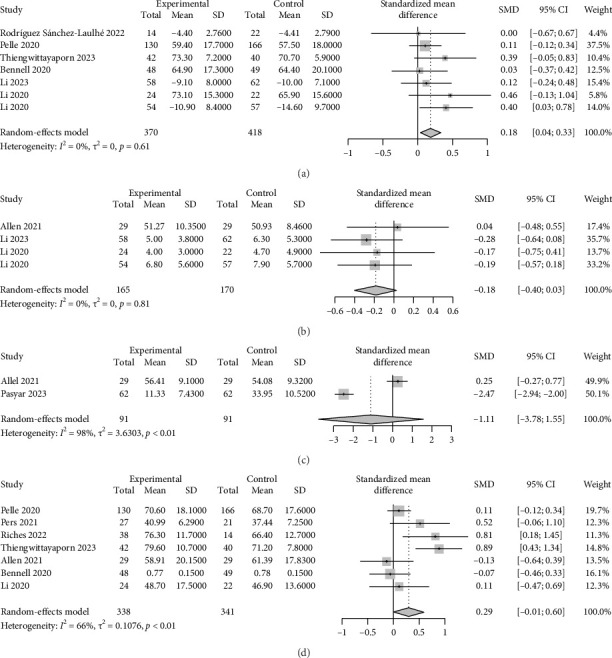
The effects of EMIs on pain, anxiety, depression, and quality of life. (a) The effect of EMIs on pain. (b) The effect of EMIs on depression. (c) The effect of EMIs on anxiety. (d) The effect of EMIs on QoL.

**Figure 3:**
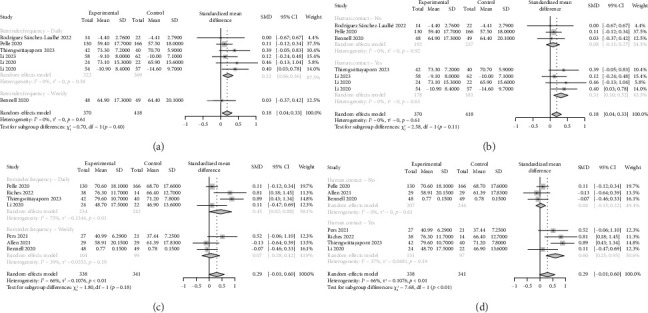
Subgroup analysis for the effects of EMIs on pain and quality of life. (a) Subgroup analysis for the effects of EMI on pain based on reminder frequency. (b) Subgroup analysis for the effects of EMI on pain based on human contact or not. (c) Subgroup analysis for the effects of EMI on QoL based on reminder frequency. (d) Subgroup analysis for the effects of EMI on QoL based on human contact or not.

Pain was evaluated using generic (e.g., visual analog scale, numeric rating scale, McGill pain questionnaire) [23, 25, 42, 45, 48] or disease-specific questionnaires [22, 30, 43, 44, 46, 47] across 11 studies. Two studies reported between-group reductions in pain [22, 23], while one study found within-group reductions after the ecological momentary intervention [44]. The overall meta-analysis revealed a trivial but statistically significant reduction in pain in the ecological momentary intervention groups compared to controls (SMD = 0.18, 95% CI: 0.04–0.33; *I*^2^ = 0%, *p*=0.61, Figure 2(a)). The result of the sensitivity analysis for pain presented in Supporting Figure 2 indicated that the results were stable. Subgroup analyses further elucidated these findings. Specifically, pain reduction was significantly greater in participants who received daily reminders (SMD = 0.21, 95% CI: 0.06–0.36; *I*^2^ = 0%, *p*=0.58), whereas there was no significant difference in pain between weekly reminder ecological momentary intervention groups and controls (SMD = 0.03, 95% CI: −0.37–0.42, Figure 3(a)). Additionally, the reduction in pain was significantly greater in participants who received ecological momentary interventions that involved human contact (SMD = 0.31, 95% CI: 0.10–0.52; *I*^2^ = 0%, *p*=0.63), but not in those who received ecological momentary interventions without human contact (SMD = 0.08, 95% CI: −0.11–0.27; *I*^2^ = 0%, *p*=0.92; Figure 3(b)).

#### 3.5.2. Depression and Anxiety

Depression was evaluated in 4 studies using the Patient Health Questionnaire-9 items [25, 47, 48] or depression domains of PROMIS-29 [24], among which 1 study observed a reduction in this outcome after the ecological momentary intervention [25]. The overall meta-analysis revealed a small but marginally significant reduction in depression scores in the ecological momentary intervention groups compared to the controls (SMD = −0.18, 95% CI: −0.40–0.03; *I*^2^ = 0%, *p*=0.81, Figure 2(b)). The result of the sensitivity analysis for depression presented in Supporting Figure 3 indicated that the results were stable. Only two studies reported changes in anxiety using anxiety domains of PROMIS-29 [24] or health anxiety inventory [26], with one study showing significantly lower anxiety scores in the ecological momentary intervention group compared to the control group [26]. However, the overall meta-analysis did not find statistically significant reductions in anxiety between the ecological momentary intervention and control groups (SMD = −1.11, 95% CI: −3.78–1.55; *I*^2^ = 98%, *p* < 0.01; Figure 2(c)). The result of the sensitivity analysis for anxiety presented in Supporting Figure 4 showed that the pooled result was the opposite after removing the study by Allen et al. [24], which may have been influenced by low participant engagement with the ecological momentary interventions.

#### 3.5.3. Quality of Life

Quality of life was assessed in 11 studies, using generic (e.g., EuroQoL 5-Dimension 3-Level questionnaire, 12-item short-form health survey) [22, 27–29, 46] or disease-specific questionnaires [24, 30, 42–44, 47]. Four studies reported between-group improvements in quality of life or specific quality-of-life domains [27–30], while two studies found within-group enhancement after the ecological momentary intervention [44]. The overall meta-analysis demonstrated a small but marginally significant improvement in quality of life favoring the ecological momentary intervention groups over controls (SMD = 0.29, 95% CI: −0.01–0.60; *I*^2^ = 66%, *p* < 0.01, Figure 2(d)). The result of the sensitivity analysis for quality of life presented in Supporting Figure 5 revealed that the overall findings were sensitive to the exclusion of the studies by Allen et al. [24] and Bennell et al. [46], which may have been influenced by low participant engagement with the ecological momentary interventions. Subgroup analysis further revealed that the improvement of quality of life was more pronounced in participants who received daily reminders (SMD = 0.45, 95% CI: 0.02–0.88; *I*^2^ = 75%, *p* < 0.01), whereas there was not any significant difference between participants in weekly reminder ecological momentary intervention groups and controls (SMD = 0.07, 95% CI: −0.28–0.42; *I*^2^ = 39%, *p*=0.19; Figure 3(c)). Additionally, the improvement in quality of life was significantly greater in participants who received ecological momentary interventions that involved human contact (SMD = 0.60, 95% CI: 0.25–0.95; *I*^2^ = 37%, *p*=0.19), but not in those who received ecological momentary interventions without human contact (SMD = 0.04, 95% CI: −0.15–0.22; *I*^2^ = 0%, *p*=0.61; Figure 3(d)).

### 3.6. Feasibility and Acceptability

The majority of studies (11 out of 16) reported data on participants' engagement with the ecological momentary interventions. Eight studies provided metrics on the percentage of app usage [22–24, 42, 44, 46–48], while three studies reported the mean duration between two app usage records [25, 28, 29]. Notably, one study found that only 50% of participants in the ecological momentary intervention group used the app [24]. In contrast, a substantially higher rate of app usage was reported by Gohir et al. (87.9%) [23] and Li et al. (83.1%) [48].

In terms of participant satisfaction, seven studies reported relevant data [22, 24, 25, 27–29, 42]. The studies by Kuusalo [27] and Riches [29] showed that 97.6% and 93.0% of participants in the ecological momentary intervention groups provided positive evaluations of the interventions, respectively. However, Pelle et al. [22] found that only 26% of participants were willing to continue using the app after the intervention ended.

Regarding the safety of the ecological momentary interventions, eight studies reported adverse [25, 28, 29, 45, 47, 48] or serious adverse events [23, 27] were observed during the interventions. However, the authors explicitly stated that these adverse events were not attributed to the ecological momentary interventions themselves (Table 1).

## 4. Discussion

### 4.1. Main Findings

To our knowledge, this was the first meta-analysis to evaluate the efficacy of ecological momentary interventions in reducing pain, improving mental health, and enhancing quality of life among individuals with rheumatic diseases. We found moderate-quality evidence that ecological momentary interventions significantly reduce pain. However, the evidence was of low or very low quality for the effects of ecological momentary interventions on mental health outcomes, and there was no statistically significant impact on depression and anxiety. For quality of life, the analysis revealed low-quality evidence that ecological momentary interventions had a marginally significant effect in improving quality of life. These findings suggest the need for more high-quality RCTs to draw robust conclusions about the benefits of ecological momentary interventions for individuals with rheumatic diseases.

### 4.2. Interpretation of Findings

#### 4.2.1. Effect of Ecological Momentary Interventions on Pain

The moderate-certainty evidence that ecological momentary interventions can significantly reduce pain among individuals with rheumatic diseases supports the use of these interventions for pain management in this population. This beneficial effect is likely attributable to the incorporation of self-management techniques or behavior change strategies in the included studies. These approaches have been considered favorable solutions for alleviating pain in chronic conditions, as they can empower participants to take personal responsibility for monitoring and managing their health status throughout diseases [49, 50]. Ecological momentary interventions that provided daily reminders had a larger effect on pain relief compared to those with weekly reminders, possibly by helping participants maintain a sustained focus on disease management and enhancing adherence [51]. Additionally, ecological momentary interventions with daily reminders can closely monitor participants' pain status and make timely adjustments to the intervention measures based on the feedback, leading to better pain relief outcomes. This is consistent with previous research, suggesting that high-frequency assessments are more effective in symptom improvement [52]. Besides, ecological momentary interventions involving human involvement, such as clinician or researcher interactions, demonstrated more significant pain-reducing effects than automated ecological momentary intervention delivery. This may be due to the development of a stronger sense of responsibility and willingness to actively participate in the intervention when participants have regular contact with healthcare providers, who can also deliver more personalized feedback and support [53–55].

#### 4.2.2. Effect of Ecological Momentary Interventions on Mental Health

Contrary to our expectations, the meta-analysis found no statistically significant effect of ecological momentary interventions on depression and anxiety among individuals with rheumatic diseases. This is noteworthy that depression and anxiety are strongly associated with psychosocial factors, such as negative emotions, low social support, and maladaptive coping mechanisms [7, 56, 57]. However, the majority of the ecological momentary interventions were primarily designed to target physical activity (*N* = 3, 60%), or pain management (*N* = 1, 20%), rather than incorporating specific modules to address the psychosocial aspects of mental health. This mismatch between the intervention contents and the intended mental health outcomes may have reduced the intervention's ability to effectively alleviate symptoms of depression and anxiety [58, 59]. The lack of effect may also be partially attributed to the opposite results reported in a single study [24]. Additionally, the very low and low certainty of the evidence, due to methodological limitations and heterogeneity across studies, may have contributed to the statistically insignificant findings on mental health outcomes.

#### 4.2.3. Effect of Ecological Momentary Interventions on Quality of Life

The results suggest that ecological momentary interventions marginally improve quality of life for individuals with rheumatic diseases, which may be primarily driven by the beneficial effect of ecological momentary interventions in reducing pain since pain is a key factor influencing quality of life [60]. Furthermore, promptly addressing any problems or challenges encountered by participants through the ecological momentary intervention platform can help resolve issues that may otherwise negatively impact their quality of life [61]. The finding that daily reminders led to greater quality-of-life benefits than weekly reminders highlights the importance of intervention intensity, as more frequent prompts can enhance patient adherence and engagement [19]. Then, regular exposure to the intervention could help foster habit formation and improve self-monitoring skills, thereby improving quality of life [19, 62]. Furthermore, ecological momentary interventions involving human involvement demonstrated stronger quality-of-life outcomes than automated ecological momentary intervention delivery. Direct engagement with healthcare providers likely enhances the clinical relevance, personalization, and accountability of the self-management strategies or behavior change techniques delivered through the ecological momentary interventions [63]. Participants may feel more supported, empowered, and confident in managing their condition when they can access expert guidance and tailored recommendations, leading to a better quality of life [58, 64].

### 4.3. Feasibility and Acceptability

The variability observed in user engagement metrics, ranging from low to high adoption rates, suggests that maintaining consistent and meaningful usage of ecological momentary interventions remains a significant challenge. Some principles, such as user-centered design and gamification, are strongly recommended for designing platforms to increase the engagement of users [65, 66]. The mixed results on participant satisfaction also highlight the importance of carefully evaluating the acceptability of ecological momentary interventions from the end-user perspective, as developing interventions that not only provide immediate utility but also foster long-term adoption and continued use will be crucial [67]. Encouragingly, the lack of safety concerns directly attributed to the ecological momentary interventions themselves is a positive indication that these digital tools can be implemented with acceptable risk profiles, supporting the continued exploration and refinement of ecological momentary interventions as adjuncts or alternatives to traditional modes of healthcare delivery.

### 4.4. Limitation

This review has several limitations. First, this study only involved 4 rheumatic diseases (rheumatoid arthritis, osteoarthritis, systemic lupus erythematosus, and juvenile idiopathic arthritis), and most of the included studies had relatively small sample sizes. Therefore, caution should be taken when generalizing the findings to other rheumatic diseases. Second, some authors did not respond to the data request emails, and some current data were not included in the analyses, which may have impacted the comprehensiveness of the analyses. Additionally, the heterogeneity in the delivery models, components, durations, and outcome measures may have introduced clinical heterogeneity, despite our efforts to apply strict inclusion criteria to reduce such heterogeneity. Finally, a majority of the included trials exhibited measurement biases and unclear selection processes. The quality of the evidence, as assessed using the GRADE criteria, ranged from very low to moderate (Supporting Table 2). Consequently, the results should be interpreted with appropriate caution.

### 4.5. Implications

This study provides supporting evidence for the potential benefits of ecological momentary interventions in improving health outcomes for individuals with rheumatic diseases. As such, healthcare providers should consider integrating ecological momentary interventions into routine care as a complementary approach to optimize existing healthcare management strategies. Also, when designing future ecological momentary interventions, research should focus on optimizing the parameter settings, such as reminder frequency, and human-interactive elements, to enhance their efficacy. Additionally, further evaluation of the effectiveness of ecological momentary interventions in different rheumatic disease subtypes is needed to strengthen the evidence base for clinical application. Despite the positive impact of ecological momentary interventions on pain and quality of life, the study findings indicate that they have not yet achieved significant improvements in mental health indicators, such as anxiety and depression. Therefore, future research should prioritize the optimization of ecological momentary intervention design and implementation to better address the mental health needs of individuals with rheumatic diseases. Furthermore, high-quality RCTs are still needed to rigorously validate the effects of ecological momentary interventions and provide more robust evidence to guide clinical practice.

## 5. Conclusion

Our review demonstrated that ecological momentary interventions can, to some extent, alleviate pain and improve quality of life among individuals with rheumatic diseases, but have not achieved significant improvements in anxiety and depression. Future research should focus on further optimizing the design and implementation of ecological momentary interventions and delving deeper into their impact on mental health. Ecological momentary interventions represent an innovative approach that deserves further promotion and application in the clinical management of rheumatic diseases.

## Figures and Tables

**Table 1:** Characteristics of the included studies.

First author, year, country	Study design and data analysis methods	Participants	Control	Outcomes (measures)	Feasibility and acceptability	Main findings
Rodríguez Sánchez-Laulhé et al. 2022 Spain [45]	RCT Intention-to-Treat	Diagnosis: Rheumatoid arthritis Sample size *N* = 36 IG = 14; CG = 22 IG: Age (*M* ± SD): 57.64 ± 7.25 Gender (female): 64.0% CG: Age (*M* ± SD): 61.86 ± 10.76 Gender (female): 59.0%	Usual care	Pain (11-point VAS)	Recruitment rate: 36/41 = 87.8% Retention rate: 30/36 = 83.0% Measurement completion rate: 92.9% of participants completed the assessment after 3 months of the intervention Adverse event participants experienced disease-associated pain flare-ups during follow-up; no adverse events related to interventions were reported throughout the trial	No time × group interaction was observed for pain (*p*=0.26)

Pelle et al. 2020 Netherlands [22]	RCT Intention-to-Treat	Diagnosis: Knee/hip osteoarthritis Sample size: *N* = 427 IG = 214; CG = 213 IG: Age (*M* ± SD): 62.1 ± 7.7 Gender (female): 68.7% CG: Age (*M* ± SD): 62.1 ± 7.0 Gender(female): 74.7%	Usual care	Health-related quality of life (EQ-5D-3L) Pain (KOOS/HOOS)	Recruitment rate: 427/692 = 61.7% Retention rate: 322/427 = 75.4% Measurement completion rate: 70.1% of participants completed the assessment after 3 months of the intervention Application engagement: (1) 171 (80%) opened the app at least once, of all participants, 151 (71%) chose at least one goal, a total of 113 (53%) participants achieved at least one goal; (2) people active with completing at least one goal (*N* = 113), the median length of use was 144 (interquartile range: 63, 173) days, with a median of 33 (interquartile range: 16, 89) logins per user Satisfaction: A total of 56 (26%) of all participants in the IG still used the app after the intervention	(1) Positive changes were found for pain between groups over 6 months (overall effect: 3.5, 95% CI: 0.9–6.0) (2) At postintervention, no significant between-group differences in health-related quality of life (overall effect: −1.7, 95% CI: −4.5 to 1.0)

Pers et al. 2021 France [28]	RCT Intention-to-Treat	Diagnosis: Rheumatoid arthritis Sample size *N* = 89 IG = 44; CG = 45 IG: Age: 19–39 (9.1%), 40–59 (47.7%), 60–75 (43.2%) Gender (female): 77.3% CG: Age: 19–39 (8.9%), 40–59 (44.4%), 60–75 (46.7%) Gender (female): 73.3%	Usual care	Health-related quality of life (SF-12)	Recruitment rate: 94/178 = 52.8% Retention rate: 89/94 = 94.7% Measurement completion rate: 93.8% of participants completed the assessment after 3 months of the intervention Application engagement: During the last 16 weeks of the study, patients in the IG returned complete information in the application (at least 75% of the items requested at least one time during the week) in 10.6 ± 4.1 weeks Satisfaction: (1) 80% of participants in the IG were eager to continue the connected monitoring; (2) 93.2% of participants in the IG would recommend the connected monitoring to their family and friends Adverse events: 26 adverse events were reported (8 in the IG); none were attributed to either of the interventions	(1) Significant improvements were observed after 6 months between the CG and IG in the SF-12 scores for role limitations due to physical health problems (IG: 67.07 ± 24.49 vs. CG: 51.60 ± 30.78, *p*=0.03) and role limitations due to emotional problems (IG: 69.79 ± 19.90 vs. CG: 55.15 ± 27.02, *p*=0.03) (2) There were significant changes from baseline to 6 months between CG and IG in the SF-12 scores for physical functioning (IG: 18.89 ± 5.12 vs. CG: 3.98 ± 5.18, *p*=0.04) and role limitations due to physical health problems (IG: 19.83 ± 4.04 vs. CG: 8.71 ± 4.16, *p*=0.05) (3) No differences were found between IG and CG in other domains of SF-12

Riches et al. 2022 United Kingdom [29]	RCT Intention-to-Treat	Diagnosis: gout Sample size *N* = 60 IG = 40; CG = 20 IG: Age (*M* ± SD): 53.0 ± 15.0 Gender (female): 8.0% CG: Age (*M* ± SD): 52.6 ± 14.1 Gender (female): 1.0%	Active control (A limited version of the application)	Health-related quality of life (EQ-5D-5L)	Recruitment rate: 60/92 = 65.2% Retention rate: 54/60 = 90.0% Measurement completion rate: 90.0% self-monitoring ongoing at 12 months Application engagement: for quality-of-life diary entries, the IG had an average number of reminders required per submission of 9.53 ± 9.25 days, compared to 17.30 ± 9.86 days in the CG Satisfaction: (1) 93% of participants in the IG group said they liked the app at 24 weeks; (2) only 30% of participants offered responses to a question about what they did not like about the approach Adverse events: 7 serious adverse events and 19 adverse events were reported (4 and 5 in the IG), and none were attributed to either of the interventions	(1) There was a statistically significant difference in the quality of life (IG: 76.30 ± 11.70 vs. CG 66.40 ± 12.70, *p*=0.011) in months 1–6 and the ability to carry out the usual activity domain (IG: 1.20 ± 0.46 vs. CG 1.78 ± 1.22, *p*=0.028) in months 7–12 (2) Significant improvements in the IG were observed from baseline to months 7–12 in the mobility domain (1.51 ± 0.66 vs. 1.24 ± 0.40, *p*=0.042), pain domain (1.91 ± 0.74 vs. 1.44 ± 0.57, *p*=0.003), and quality of life (74.50 ± 16.40 vs. 83.40 ± 11.10, *p*=0.009). (3) There were no significant differences between the CG and IG in months 1–6 and months 7–12 for other subscales

Thiengwittayaporn et al. 2023 Thailand [30]	RCT Intention-to-Treat	Diagnosis: Knee osteoarthritis Sample size *N* = 82 IG = 42; CG = 40 IG: Age (*M* ± SD): 62.2 ± 6.8 Gender (female): 85.7% CG: Age (*M* ± SD): 63.0 ± 19.7 Gender (female): 92.5%	Active control (handout self-directed exercise)	Health-related quality of life and pain (KOOS)	Recruitment rate: 89/106 = 84.0% Retention rate: 82/89 = 92.1% Measurement completion rate: 95.2% completed the assessment after 4 weeks of the intervention	(1) Significant improvements were observed after 4 weeks between the CG and IG in the quality of life (IG: 79.6 ± 10.7 vs. CG: 71.2 ± 7.8, *p*=0.009) (2) Significant improvements in the IG were observed from baseline to 4 weeks in quality of life (69.5 ± 6.2 vs. 79.6 ± 10.7, *p* < 0.001) (3) No statistically significant changes were found in pain between groups (IG: 73.3 ± 7.2 vs. CG: 70.7 ± 5.9, *p*=0.279), nor within the IG from baseline to 4 weeks (72.0 ± 6.8 vs. 73.3 ± 7.2, *p*=0.089)

Allen et al. 2021 USA [24]	Pilot RCT Intention-to-Treat	Diagnosis: systemic lupus erythematosus Sample size *N* = 60 IG = 30; CG = 30 IG: Age (*M* ± SD): 51 ± 14 Gender (female): 93.0% CG: Age (*M* ± SD): 47 ± 12 Gender (female): 97.0%	Waitlist	Depression (PROMIS-29) Anxiety (PROMIS-29) Quality of life (LupusPRO)	Recruitment rate: 60/85 = 70.6% Retention rate: 58/60 = 96.7% Measurement completion rate: 100% of participants completed the follow-up survey Application engagement: 50% of participants used the app Satisfaction: (1) the app received a median rating of 8.0 for helpfulness on a scale of 0–10 among program users; (2) participants consistently expressed appreciation for the convenience of the online format; (3) some participants mentioned difficulties in relating to certain aspects of the app	At postintervention, no significant between-group differences in anxiety (mean change: 0.4 ± 11.2 vs. 1.4 ± 9.3, effect size: 0.097, 95% CI: −0.409–0.604), depression (mean change: −0.6 ± 9.0 vs. −3.4 ± 8.6, effect size: −0.318, 95% CI: −0.827 to 0.191), and health-related quality of life (mean change: 1.4 ± 12.0 vs. 2.0 ± 13.3, effect size: 0.047, 95% CI: −0.459–0.554)

Kloek et al. 2018 Netherlands [44]	RCT Intention-to-Treat	Diagnosis: hip/knee osteoarthritis Sample size *N* = 208 IG = 109; CG = 99 IG: Age (*M* ± SD): 63.8 ± 8.5 Gender (female): 67.9% CG: Age (*M* ± SD): 62.3 ± 8.9 Gender (female): 67.7%	Active control (usual physical therapy)	Health-related quality of life and pain (KOOS)	Recruitment rate: 208/246 = 84.6% Retention rate: 176/208 = 84.6% Measurement completion rate: 89% of participants completed the questionnaire at the end of the intervention Application engagement: (1) at the 3-month mark, the average system usability score of the 85 individuals who responded was 73.1 (SD = 18.6), indicating a Grade B usability rating; (2) adherence data were available for 90 out of 109 participants. Among these participants, 73 (81.1%) completed at least 8 out of 12 modules, thus meeting the criteria for being classified as “adherent”	(1) Within the IG, significant improvements were observed in the quality of life and pain at the 3-month point (45.0 vs. 49.1, *p*=0.02; 50.4 vs. 55.8, *p* < 0.01) and 12-month time point (45.0 vs. 52.5, *p* < 0.01; 50.4 vs. 65.9, *p* < 0.01) (2) There were no significant statistical differences in quality of life and pain between the two groups at 3 months and 12 months

Kuusalo et al. 2020 Finland [27]	RCT Intention-to-Treat	Diagnosis: Rheumatoid arthritis Sample size *N* = 162 IG = 82; CG = 80 IG: Age: 54 ± 13 Gender (female): 71.0% CG: Age: 59 ± 14 Gender (female): 70.0%	Usual care	Health-related quality of life (SF-36)	Recruitment rate: none reported Retention rate: 162/166 = 97.6% Measurement completion rate: 97.6% of participants completed the follow-up questionnaire Satisfaction: (1) the vast majority of patients (97.6%) provided positive evaluations of the system; (2) all patients (100%) indicated they would recommend the SMS monitoring for other rheumatoid arthritis patients; (3) 94% of patients found the monitoring messages technically easy to answer, and over 80% felt secure and satisfied with their treatment. However, 25% of the patients reported finding the self-assessment of disease activity using the patient global assessment somewhat difficult or difficult. Adverse events: 2 serious adverse events and 97 adverse events were reported (1 and 51 in the IG); none were attributed to either of the interventions	(1) Improvement in the SF-36 dimension of physical function was greater in the IG than in the CG (*p*=0.042) (2) Changes in physical and mental summary components did not differ between the randomization groups

Bennell et al. 2020 Australia [46]	RCT Intention-to-Treat	Diagnosis: Knee osteoarthritis Sample size *N* = 110 IG = 56; CG = 54 IG: Age: 61.7 ± 6.7 Gender (female)62.0% CG: Age: 62.9 ± 6.8 Gender (female)72.0%	Active control (structured home exercise program without SMS contact)	Health-related quality of life (AQoL) Pain (KOOS)	Recruitment rate: 110/128 = 85.9% Retention rate: 99/110 = 90.0% Measurement completion rate: 85.7% of participants completed a 24-week assessment Application engagement: the mean reply rate per participant to self-reporting home exercise sessions was 66%	There was no evidence of between-group differences in health-related quality of life and pain (mean difference: −0.01, 95% CI: −0.06 to 0.04, *p*=0.68; mean difference: 1.3, 95% CI: −4.6 to 7.3, *p*=0.66)

Li et al. 2023 Canada [25]	RCT Intention-to-Treat	Diagnosis: Rheumatoid arthritis Sample size *N* = 131 IG = 65; CG = 66 IG: Age: 54.8 ± 13.1 Gender (female): 92.3% CG: Age: 56.9 ± 13.2 Gender (female): 90.9%	Waitlist control	Depression (PHQ-9) Pain (McGill Pain Questionnaire)	Recruitment rate: 131/140 = 93.6% Retention rate: 120/131 = 91.6% Measurement completion rate: 89.2% of participants completed the 27-week follow-up questionnaire Application engagement: (1) during the intervention period, participants used the app for an average of 150.1 days (SD 44.5) and recorded information on the app 22.8 times (SD 26.9); (2) the mean duration between two records was 12.1 days (SD 9.4) Satisfaction: participants appeared to continue using the app for tracking their health in weeks 27–52 after the counseling calls ended Adverse events: 3 adverse events in the IG were reported, but none were attributed to either of the interventions	(1) A favorable intervention effect was found in depression (adjusted coefficient: −1.6, 95% CI -2.9 to −0.3, *p* < 0.05) (2) No significant effect was found in pain (adjusted coefficient: −1.1, 95% CI -3.0 to 0.7)

Li et al. 2020 Canada [47]	RCT Intention-to-Treat	Diagnosis: Knee osteoarthritis Sample size *N* = 51 IG = 26; CG = 25 IG: Age: 65.0 ± 8.0 Gender (female):89.0% CG: Age: 64.8 ± 9.0 Gender (female):76.0%	Waitlist control	Health-related quality of life and pain (KOOS) Depression (PHQ-9)	Recruitment rate: 51/57 = 89.5% Retention rate: 48/51 = 94.1% Measurement completion rate: 92.3%, participants completed the 13-week follow-up questionnaire Application engagement: 81% of participants used the Fitbit app Adverse events: 10 adverse events were reported (8 in the IG); none were attributed to either of the interventions	No statistical significance was found for results from health-related quality of life (mean difference: 1.4, 95% CI: −5.0 to 7.9, *p*=0.50), pain (mean difference: 2.5, 95% CI: −4.2 to 9.5, *p*=0.49), and depression (mean difference: −0.4, 95% CI: −1.7 to 0.8, *p*=0.47)

Li et al. 2020 Canada [48]	RCT Intention-to-Treat	Diagnosis: systemic lupus erythematosus or rheumatoid arthritis Sample size *N* = 118 IG = 59; CG = 59 IG: Age: 53.5 ± 14.7 Gender (female): 86.4% CG: Age: 53.1 ± 12.6 Gender (female): 91.5%	Waitlist control	Depression (PHQ-9) Pain (McGill Pain Questionnaire)	Recruitment rate: 118/121 = 97.5% Retention rate: 113/118 = 95.8% Measurement completion rate: 94.9%, participants completed the 9-week follow-up questionnaire Application engagement: 83.1% of participants used the Fitbit app Adverse events: 23 adverse events were reported (13 in the IG); none were attributed to either of the interventions	No statistical significance was found for results from depression (mean difference: −0.31, 95% CI: −1.87 to 1.24) and pain (mean difference: −2.29, 95% CI: −4.66 to 0.08)

Khan et al. 2020 USA [43]	RCT Intention-to-Treat	Diagnosis: systemic lupus erythematosus Sample size *N* = 46 IG = 25; CG = 21 IG: Age: 44 (33, 51) Gender (female): 96.0% CG: Age: 42 (36, 50) Gender (female): 95.0%	Usual care	Health-related quality of life and pain (LupusQoL)	Recruitment rate: none reported Retention rate: 47/50 = 94.0% Measurement completion rate: 64% of participants completed the follow-up questionnaire	(1) Within the IG, a significant improvement over baseline was noted for the burden to others dimension (16.7 vs. 25.0, *p*=0.02) and fatigue dimension (25.0 vs. 62.5, *p*=0.007) (2) There was no evidence of between-group differences in other dimensions of health-related quality of life and pain

Lalloo et al. 2020 Canada [42]	RCT Intention-to-Treat	Diagnosis: Juvenile idiopathic arthritis Sample size *N* = 60 IG = 29; CG = 31 IG: Age: 14.9 ± 1.7 Gender (female): 79.3% CG: Age: 15.1 ± 1.6 Gender (female): 77.4%	Active control (a limited version of the app)	Health-related quality of life (PedsQL3.0 arthritis module) Pain (numerical rating scale)	Recruitment rate: 60/73 = 82.2% Retention rate: 52/60 = 86.7% Measurement completion rate: 86% of participants completed the follow-up questionnaire Application engagement: (1) the mean number of completed daily check-ins was 29.6 (SD 14.5) for IG and 29.0 (SD 11.7) for CG; (2) 64% of participants in the IG and 79% of participants in the CG accessed the history function at least once during the 55-day study period; Satisfaction: (1) the mean acceptability scores were 34.7 (SD 4.9) and 34.6 (SD 3.7) for IG and CG participants, respectively; (2) a majority of intervention participants (57%) were willing to use their assigned app for longer than 8 weeks	There were no significant changes in health-related quality of life and pain

Gohir et al. 2021 United Kingdom [23]	RCT Intention-to-Treat	Diagnosis: Knee osteoarthritis Sample size *N* = 105 IG = 48; CG = 57 IG: Age: 65.2 ± 9.7 Gender (female): 70.8% CG: Age: 68.0 ± 8.6 Gender (female): 64.9%	Usual care	Pain (numerical rating scale)	Recruitment rate:146/551 = 26.5% Retention rate: 105/146 = 71.9% Measurement completion rate: 71.6% of participants completed the follow-up questionnaire Application engagement: the mean (SD) adherence with the internet-based exercise program was 87.9% (14.3%) of sessions completed Adverse events: No serious adverse events were reported in any of the study groups	(1) The IG showed a greater decrease in pain score from baseline to 6 weeks compared with the CG (between-group difference: −1.5, 95% CI: −2.2 to −0.8, *p* < 0.001) (2) Between baseline and the 6-week follow-up, there was a statistically significant improvement in pain scores in the IG (mean change: −1.8, 95% CI: −2.4 to −1.3, *d* = −0.83) but not in the CG (mean change: −0.3, 95% CI: −0.8 to 0.2, *d* = −0.2)

Pasyar et al. 2023 Iran [26]	RCT Intention-to-Treat	Diagnosis: systemic lupus erythematosus Sample size *N* = 124 IG = 62; CG = 62 IG: Age: 44.2 ± 9.7 Gender (female): 96.8% CG: Age: 48.0 ± 8.9 Gender (female): 90.3%	Usual care	Health anxiety (health anxiety inventory)	Recruitment rate: 124/140 = 88.6% Retention rate: 124/124 = 100.0% Measurement completion rate: 100.0% of participants completed the follow-up questionnaire	(1) After the intervention, the average health anxiety scores in the IG were significantly lower than in the CG (IG: 11.33 ± 7.43 vs. CG: 33.95 ± 10.52, *p* < 0.001) (2) Comparing the mean scores in IG before and after the intervention, the Wilcoxon test revealed a statistically significant difference toward health anxiety reduction (36.77 ± 9.43 vs. 11.33 ± 7.43, *p* < 0.001), but no significant change was seen in the CG (36.22 ± 9.08 vs. 33.95 ± 10.52, *p*=0.06)

**Table 2:** Characteristics of the ecological momentary interventions.

First author, year, country	Reminder frequency and duration	Ecological momentary intervention content	Intervention response generation	Human contact
Rodríguez Sánchez-Laulhé et al. 2022, Spain [45]	A daily reminder for 3 months	(1) Participants were provided exercise therapy training, delivered with explanatory videos	AI-based	No
		(2) Participants are required to record their pain intensity before and after each exercise session, and the system automatically adjusts the load and intensity of the exercises accordingly		
		(3) The app featured an exercise diary function that visualized the progress in the planned treatment protocol and the evolution of pain intensity, as well as provided recommendations on joint protection, diet, and other self-management strategies		

Pelle et al. 2020, Netherlands [22]	A daily reminder for 3 months	(1) The app provided an educational library with information on osteoarthritis, its treatment modalities, and healthy lifestyle advice. It also includes an exercise library with 10 osteoarthritis-specific exercises	AI-based	No
		(2) Participants received a daily push notification to remind them of their chosen goal, combined with an interesting fact or answer to a frequently asked question		
		(3) Using machine learning, the app proposed new goals tailored to the participant's profile and previous goal selection behavior		

Pers et al. 2021, France [28]	Weekly reminder for 6 months	(1) Participants were prompted with weekly reminders to complete a self-questionnaire, and the investigative team received monthly biological data	Rule-based	Yes, one clinical case manager was automatically notified of any problems experienced by the patient and would advise on appropriate measures for patient care
		(2) The clinical case manager was automatically alerted to any problems encountered by the patient, enabling them to promptly address and optimize patient care		
		(3) The investigative team analyzed the data to determine the requirement for a physical or phone call visit		

Rich et al. 2022, United Kingdom [29]	A daily reminder for 24 weeks	(1) Users were prompted with daily reminders to record gout flares, quality-of-life diaries, and urate diaries	Rule-based	Yes, the research team optimized the medication regimen based on the data reported by patients
		(2) Users with a urate level exceeding 0.30 mmol/L were prompted to perform a self-test every 2 weeks		
		(3) The research team reviewed the results and made appropriate actions to optimize treatment based on the findings		

Thiengwittayaporn et al. 2023, Thailand [30]	A daily reminder for 4 weeks	(1) Participants underwent an adaptive assessment test on the mobile application to evaluate their symptom status	AI-based	Yes, the doctor exchanged information with participants and was involved in the adaptive assessment
		(2) Based on the decision tree classification results, the system recommended appropriate exercise types and number of sets and provided foundational knowledge for osteoarthritis symptom management		
		(3) Healthcare professionals dynamically assessed the stages of each patient's knee osteoarthritis, closely monitored disease progression, and offered personalized solutions to promote physical therapy and rehabilitation exercises		

Allen et al. 2021, USA [24]	Weekly reminder for 8 weeks	(1) Participants were instructed to complete one module per week in the app. Each module comprised instructional content and interactive exercises designed to enhance coping skills	AI-based	No
		(2) The modules and features of the app were personalized according to participants' progress and responses throughout the program		

Kloek et al. 2018, Netherlands [44]	Weekly reminder for 12 weeks	(1) Participants received automated notifications of new tasks weekly and were required to report the status of their task completion at the end of each week	Rule-based	No
		(2) Participants received automatically generated customized feedback based on their responses		

Kuusalo 2020, Finland [27]	13 text messages at 1–2 weeks of intervals during 24 weeks	(1) The monitoring system employed automated text messages to actively track participants' medication use and disease status	Rule-based	Yes, the nurse and physician were involved in addressing potential problems
		(2) Predefined cutoff limits for disease activity were established to detect potential problems early, prompting the system to notify the treating clinic, which would then contact the patient to address any issues		

Bennell et al. 2020, Australia [46]	Weekly reminder for 24 weeks	(1) Participants were prompted by automated text messages to self-report the number of home exercise sessions completed in the preceding week	Rule-based	No
		(2) The system provided tailored feedback and suggestions based on the participant's adherence level, in addition to delivering regular motivational messages and special occasion messages to enhance engagement		

Li et al. 2023, Canada [25]	Daily reminder for 26 weeks	(1) Participants attended a 2-h session that included group-based education on physical activity and individual counseling with a physiotherapist trained in motivational interviewing	Rule-based	Yes, a physiotherapist reviewed participants' data to continuously optimize and personalize their goals
		(2) Participants were provided with a Fitbit wearable device and the OPERAS app to track their daily physical activity, disease activity, depression, and treatment use		
		(3) Physiotherapists utilized the patient-generated data to identify barriers and solutions to help participants achieve their predefined physical activity and self-management goals		

Li et al. 2020, Canada [47]	Daily use for 12 weeks	(1) Participants were provided with a Fitbit wearable device and the FitViz app to track their daily physical activity	Rule-based	Yes, the physiotherapist regularly reviewed participants' progress data, then provided personalized feedback and recommendations
		(2) Physiotherapists remotely reviewed participants' progress on FitViz and counseled them to modify their physical activity goals via 4 biweekly phone calls		

Li et al. 2020, Canada [48]	Daily use for 8 weeks	(1) Based on the participant's goal, the physiotherapists set the physical activity parameters on FitViz to match the individual's activity plan, providing automated personalized feedback on goal attainment	Rule-based	Yes, the physiotherapist regularly reviewed participants' progress data, then provided personalized feedback and recommendations
		(2) Participants were provided with a Fitbit wearable device and the FitViz app to facilitate daily tracking of their physical activity		
		(3) Physiotherapists remotely reviewed participants' progress on FitViz and then conducted biweekly phone counseling sessions to help the participants modify their physical activity goals as needed		

Khan et al. 2020, USA [43]	Daily use for 16 weeks	(1) Participants were reminded to record their daily lifestyle activities and symptoms	AI-based	Yes, health coaches view the data in the portal and recommend interventions
		(2) Based on the software-generated insights from the self-tracking data, the health coaches recommended tailored dietary, environmental, and lifestyle interventions		
		(3) Participants were also provided broader education on nutrition, stress management, and other lifestyle factors, empowering them to make informed decisions and adopt healthier habits to support their overall well-being		

Lalloo et al. 2020, Canada [42]	Daily use for 8 weeks	(1) Participants utilized the app daily to record their pain intensity, sleep, mood, and other symptom data, identifying trends in their conditions	AI-based	
		(2) The app proactively provided users with relevant content, including disease education and symptom management techniques, based on their individual needs and reported data		
		(3) The application's community forum feature allowed users to share coping strategies and offer mutual support to one another		

Gohir et al. 2021, United Kingdom [23]	Daily use for 6 weeks	(1) Participants were provided with a digital program that delivered daily exercise routines and informative educational materials	Rule-based	Yes, a physiotherapist provided the necessary support (online or by phone)
		(2) The program dynamically adjusted the complexity, load, and difficulty of the exercises based on each participant's feedback to optimize the exercise regimen for each individual's abilities and progression		
		(3) To ensure participants had understood the key messages, each educational session was followed by a quiz		

Pasyar et al. 2023, Iran [26]	Daily use for 8 weeks	(1) Participants were given daily supportive counseling, including motivational, educational, spiritual, and psychological aspects	Rule-based	Yes, the research team would revise the program content based on responses from participants
		(2) At the end of each weekly counseling phase, the research team reviewed participants' input and, if necessary, made adjustments to the program content for the following week		

## Data Availability

The data that support the findings of this study are available from the corresponding author upon reasonable request.

## Supporting Information

Supporting Table 1. The search strategy sample—Embase.

Supporting Figure 1. The risk of bias assessment.

Supporting Table 2. The details of the GRADE evaluation.

Supporting Figure 2. The result of the sensitivity analysis for pain.

Supporting Figure 3. The result of the sensitivity analysis for depression.

Supporting Figure 4. The result of the sensitivity analysis for anxiety.

Supporting Figure 5. The result of the sensitivity analysis for quality of life.
