# Supplementary material for: Effectiveness of Ecological Momentary Interventions on Pain, Mental Health, and Quality of Life in Individuals With Rheumatic Diseases: A Systematic Review and Meta-Analysis of Randomized Controlled Trials
Source: J Nurs Manag. 2025 Jul 29;2025:9923240. doi: 10.1155/jonm/9923240 (PMC12324918; doi:10.1155/jonm/9923240)
Supplement: Supporting Information — Additional supporting information can be found online in the Supporting Information section. [file 9923240.f1.docx]

**Supplemental Materials**

**Supplementary Table 1.** The search strategy sample - Embase

**Supplementary Figure 1.** The risk of bias assessment

**Supplementary Table 2.** The details of the GRADE evaluation

**Supplementary Figure 2.** The result of the sensitivity analysis for pain

**Supplementary Figure 3.** The result of the sensitivity analysis for depression

**Supplementary Figure 4.** The result of the sensitivity analysis for anxiety

**Supplementary Figure 5.** The result of the sensitivity analysis for quality of life

| **Supplementary Table 1.** Search strategy sample - Embase | |
| --- | --- |
| Search No. | Index terms and Keywords |
| 1 | rheumatic disease.ab,ti. |
| 2 | rheumatism.ab,ti. |
| 3 | rheum*.ab,ti. |
| 4 | rheumatology.ab,ti. |
| 5 | rheumatic.ab,ti. |
| 6 | lupus.ab,ti. |
| 7 | spondylitis, ankylosing.ab,ti. |
| 8 | arthritis, rheumatoid.ab,ti. |
| 9 | arthritis, psoriatic.ab,ti. |
| 10 | scleroderma, systemic.ab,ti. |
| 11 | spondylo*.ab,ti. |
| 12 | sjogren syndrome.ab,ti. |
| 13 | osteoarthritis.ab,ti. |
| 14 | arthritis, juvenile.ab,ti. |
| 15 | gout.ab,ti. |
| 16 | 1 or 2 or 3 or 4 or 5 or 6 or 7 or 8 or 9 or 10 or 11 or 12 or 13 or 14 or 15 |
| 17 | ecological momentary intervention.ab,ti. |
| 18 | ecological momentary assessment.ab,ti. |
| 19 | ecological momentary interventions.ab,ti. |
| 20 | ecological momentary assessments.ab,ti. |
| 21 | mobile phone.ab,ti. |
| 22 | text messag*.ab,ti. |
| 23 | short message system.ab,ti. |
| 24 | wearable.ab,ti. |
| 25 | personal digital assistant.ab,ti. |
| 26 | smartphone.ab,ti. |
| 27 | ehealth.ab,ti. |
| 28 | mhealth.ab,ti. |
| 29 | digital health.ab,ti. |
| 30 | ambulatory assessment.ab,ti. |
| 31 | ambulatory monitoring.ab,ti. |
| 32 | real time data capture.ab,ti. |
| 33 | app.ab,ti. |
| 34 | digital intervention.ab,ti. |
| 35 | telehealth.ab,ti. |
| 36 | telemedicine.ab,ti. |
| 37 | internet-based intervention.ab,ti. |
| 38 | mobile applications.ab,ti. |
| 39 | ema.ab,ti. |
| 40 | emi.ab,ti. |
| 41 | 17 or 18 or 19 or 20 or 21 or 22 or 23 or 24 or 25 or 26 or 27 or 28 or 29 or 30 or 31 or 32 or 33 or 34 or 35 or 36 or 37 or 38 or 39 or 40 |
| 42 | randomized controlled trial.ab,ti. |
| 43 | randomized controlled trials.ab,ti. |
| 44 | randomized.ab,ti. |
| 45 | randomly.ab,ti. |
| 46 | rct.ab,ti. |
| 47 | 42 or 43 or 44 or 45 or 46 |
| 48 | 16 and 41 and 47 |

| 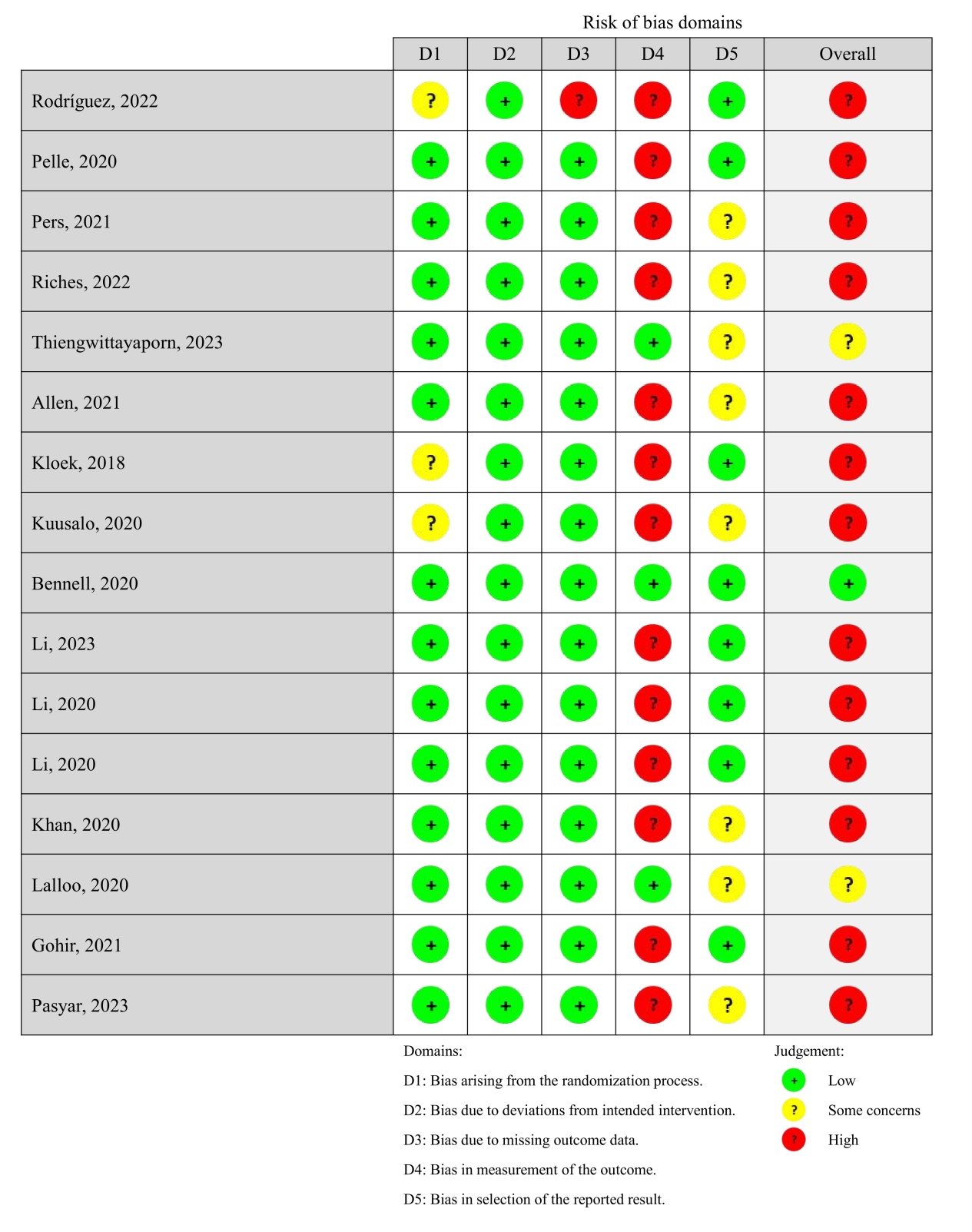 |
| --- |
| **Supplementary Figure 1 the risk of bias assessment** |

| **Supplementary Table 2. The details of the GRADE evaluation** | | | | | | | | | | | |
| --- | --- | --- | --- | --- | --- | --- | --- | --- | --- | --- | --- |
| **Certainty assessment** | | | | | | | **No. of patients** | | **Effect** | **Certainty** | **Importance** |
| **No. of studies** | **Study design** | **Risk of bias** | **Inconsistency** | **Indirectness** | **Imprecision** | **Other considerations** | **Intervention** | **Control** | **Absolute**  **(95% CI)** |  |  |
| **Pain** | | | | | | | | | | | |
| 7 | RCT | Serious^a^ | Not serious^b^ | Not serious^c^ | Not serious^d^ | None | 370 | 418 | SMD: 0.18  (0.04 to 0.33) | ⨁⨁⨁◯  Moderate | IMPORTANT |
| **Quality of life** | | | | | | | | | | | |
| 7 | RCT | Serious^a^ | Serious^e^ | Not serious^c^ | Serious^f^ | None | 338 | 341 | SMD: 0.29  (-0.01 to 0.60) | ⨁◯◯◯  Very low | IMPORTANT |
| **Depression** | | | | | | | | | | | |
| 4 | RCT | Serious^a^ | Not serious^b^ | Not serious^c^ | Serious^f^ | None | 165 | 170 | SMD: -0.18  (-0.40 to 0.03) | ⨁⨁◯◯  Low | IMPORTANT |
| **Anxiety** | | | | | | | | | | | |
| 2 | RCT | Serious^a^ | Serious^e^ | Not serious^c^ | Serious^f^ | None | 91 | 91 | SMD: -1.11  (-3.78 to 1.55) | ⨁◯◯◯  Very low | IMPORTANT |

| 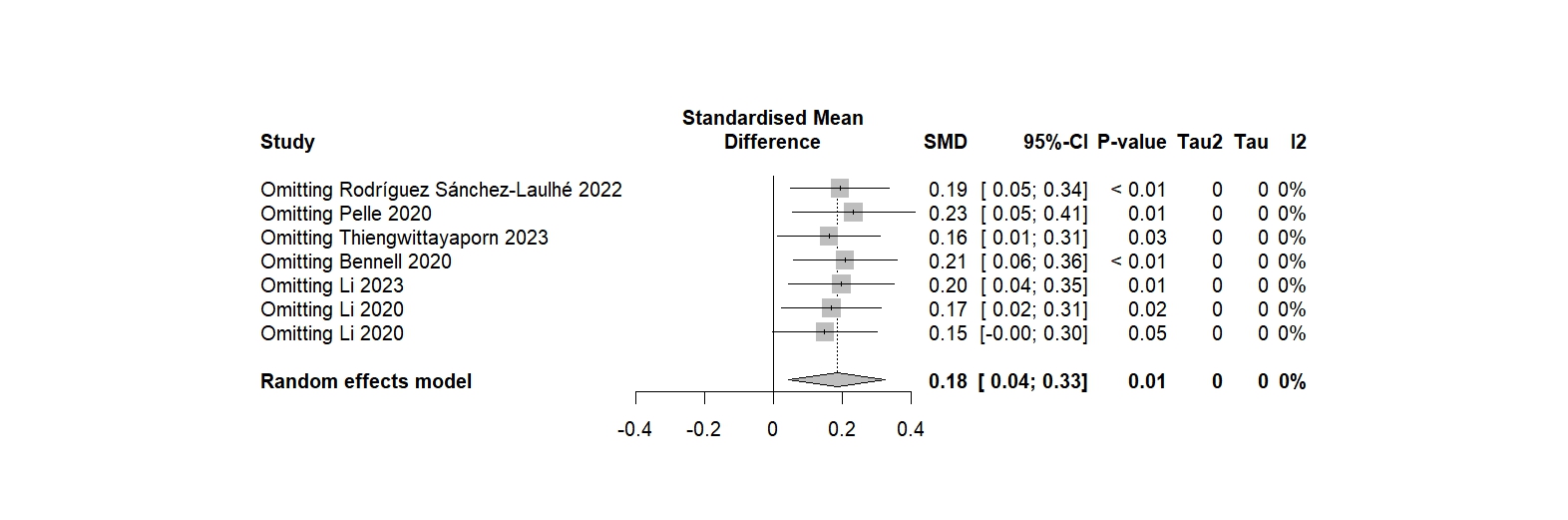 |
| --- |
| **Supplementary Figure 2 the result of the sensitivity analysis for pain** |

| 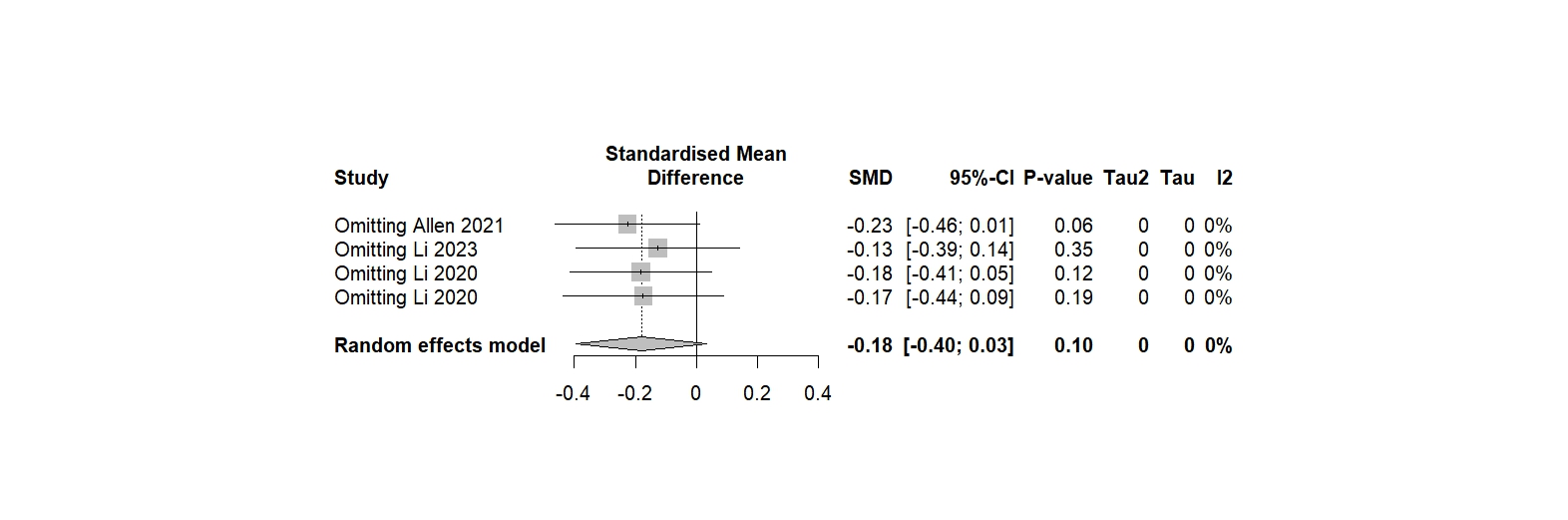 |
| --- |
| **Supplementary Figure 3 the result of the sensitivity analysis for depression** |

| 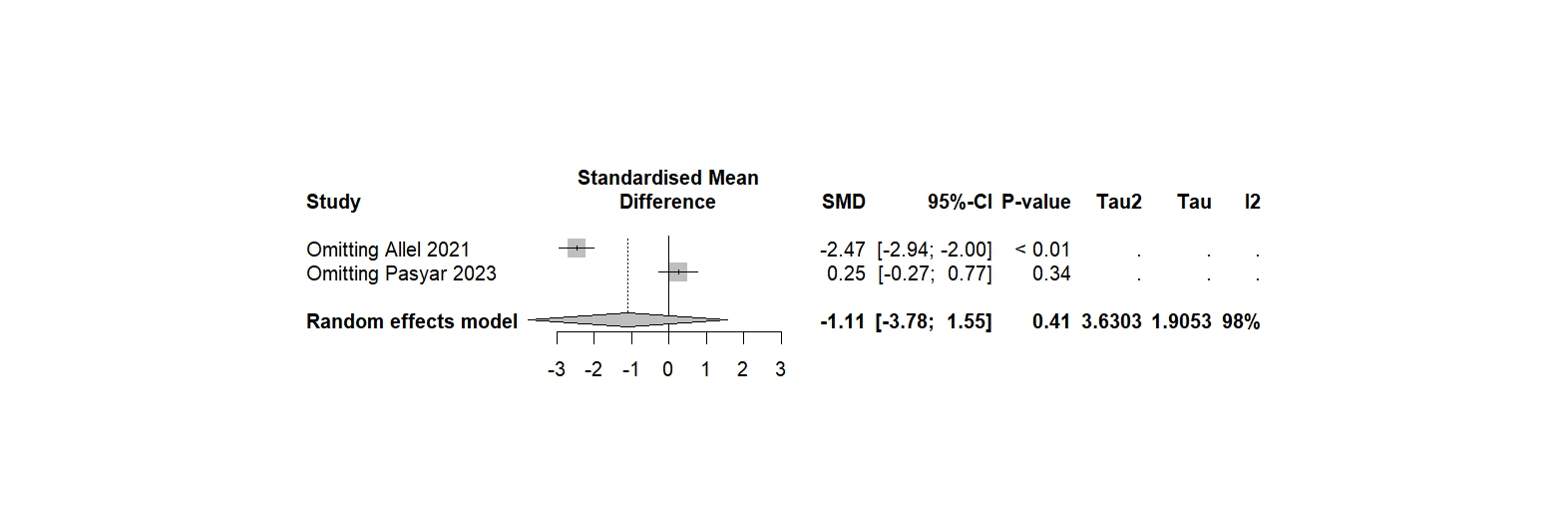 |
| --- |
| **Supplementary Figure 4 the result of the sensitivity analysis for anxiety** |

| 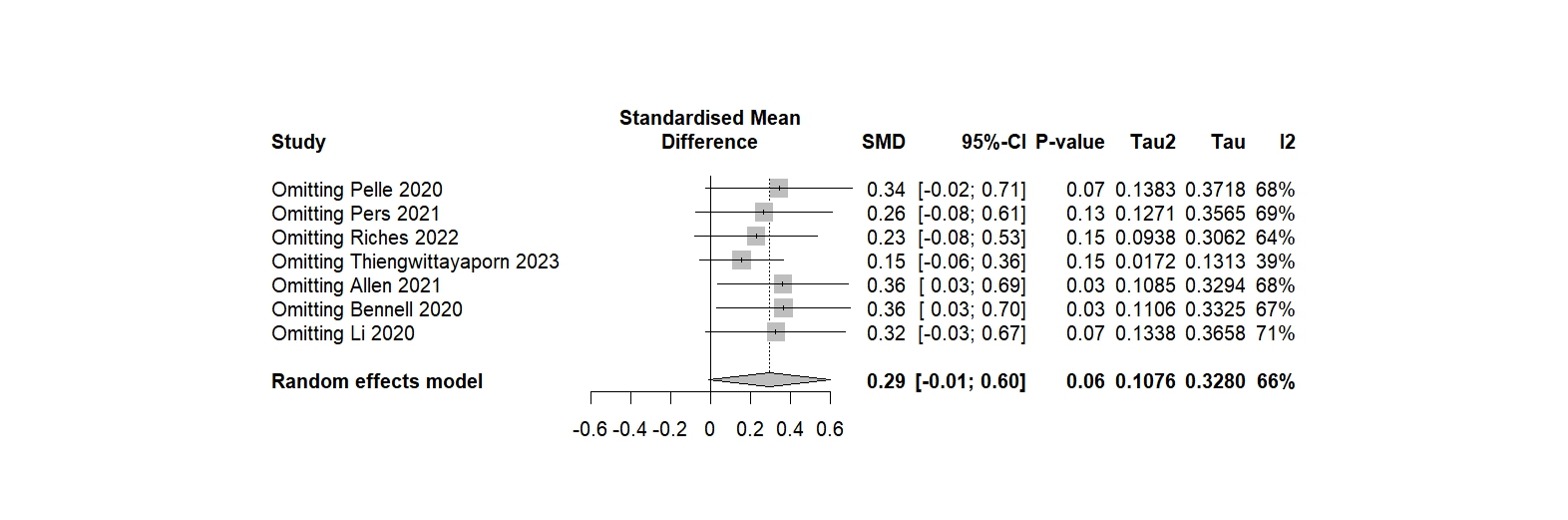 |
| --- |
| **Supplementary Figure 5 the result of the sensitivity analysis for quality of life** |
